# Innovation Deimplementation in Emergency Departments During the COVID-19 Pandemic: Qualitative Study of Clinicians’ Decision-Making

**DOI:** 10.2196/82088

**Published:** 2026-05-22

**Authors:** Shreya Huilgol, Nabeel Qureshi, Carl Berdahl, Catherine Cohen, Peter Mendel, Shira Fischer

**Affiliations:** 1 Department of Health Policy and Management, University of California, Los Angeles, 650 Charles E Young Dr S, Los Angeles, CA, 90095, United States, 1 (310) 825-6381; 2 RAND, Santa Monica, CA, United States; 3 Cedars Sinai Medical Center, Los Angeles, CA, United States; 4 RAND, Boston, MA, United States

**Keywords:** deimplementation, COVID-19 pandemic, innovation, emergency department, decision-making

## Abstract

**Background:**

During a public health emergency, emergency department (ED) clinicians can improve care delivery if they identify and adopt innovations that are safe and effective. However, little is known about the factors that impact ED clinicians’ decision-making around using or discontinuing innovations when evidence-based information is limited.

**Objective:**

The goal of this study was to understand the processes and factors that led ED clinicians to discontinue (deimplement) the use of COVID-19 care innovations.

**Methods:**

This is a qualitative study using semistructured focus groups with ED clinicians from 8 hospitals across the United States. Hospitals were purposively sampled and recruited to capture a diversity of perspectives based on location, facility type (academic or community hospital), rurality (urban or rural), and safety-net status. In this study, 17 physicians, 7 advanced practice providers, 18 nurses, and 7 respiratory therapists participated. We utilized both inductive and deductive techniques to perform content and thematic analysis of transcripts.

**Results:**

Clinicians shared that their own experiences (eg, direct observation of patient outcomes), contextual factors, and emerging research evidence contributed heavily to decisions about deimplementing innovations during the COVID-19 pandemic. Processes related to discontinuing innovations depended on leadership guidance and collaboration among colleagues. However, in some cases, there were no official processes to discontinue innovations, and innovations were passively deimplemented.

**Conclusions:**

Decision-making regarding the discontinuation of innovation in ED settings during the COVID-19 pandemic differed from routine conditions due to the lack of information and the rapid evolution of evidence within a short period of time. The level of evidence required to implement and deimplement innovations was significantly lower. Our findings indicate that factors influencing deimplementation during a public health emergency were highly localized and were treated similarly to pilot tests of new innovations. Future work is necessary to develop mechanisms for implementing promising innovations during evolving public health emergencies and monitoring their effectiveness and safety after implementation, enabling evidence-based decisions about whether to continue implementation or proceed with deimplementation.

## Introduction

During the COVID-19 pandemic, emergency department (ED) clinicians influenced patient health outcomes by rapidly identifying and implementing care innovations to manage and treat patients with COVID-19. We define care innovations as “new or improved health policies, systems, products and technologies, and services and delivery methods that improve people’s health.” [1] Examples of care innovations used in EDs during COVID-19 included utilizing prone positioning [2], high-flow oxygen [3], telemedicine [4,5], and intubation boxes [6], as well as reusing personal protective equipment (PPE) [7].

**Figure 1.**
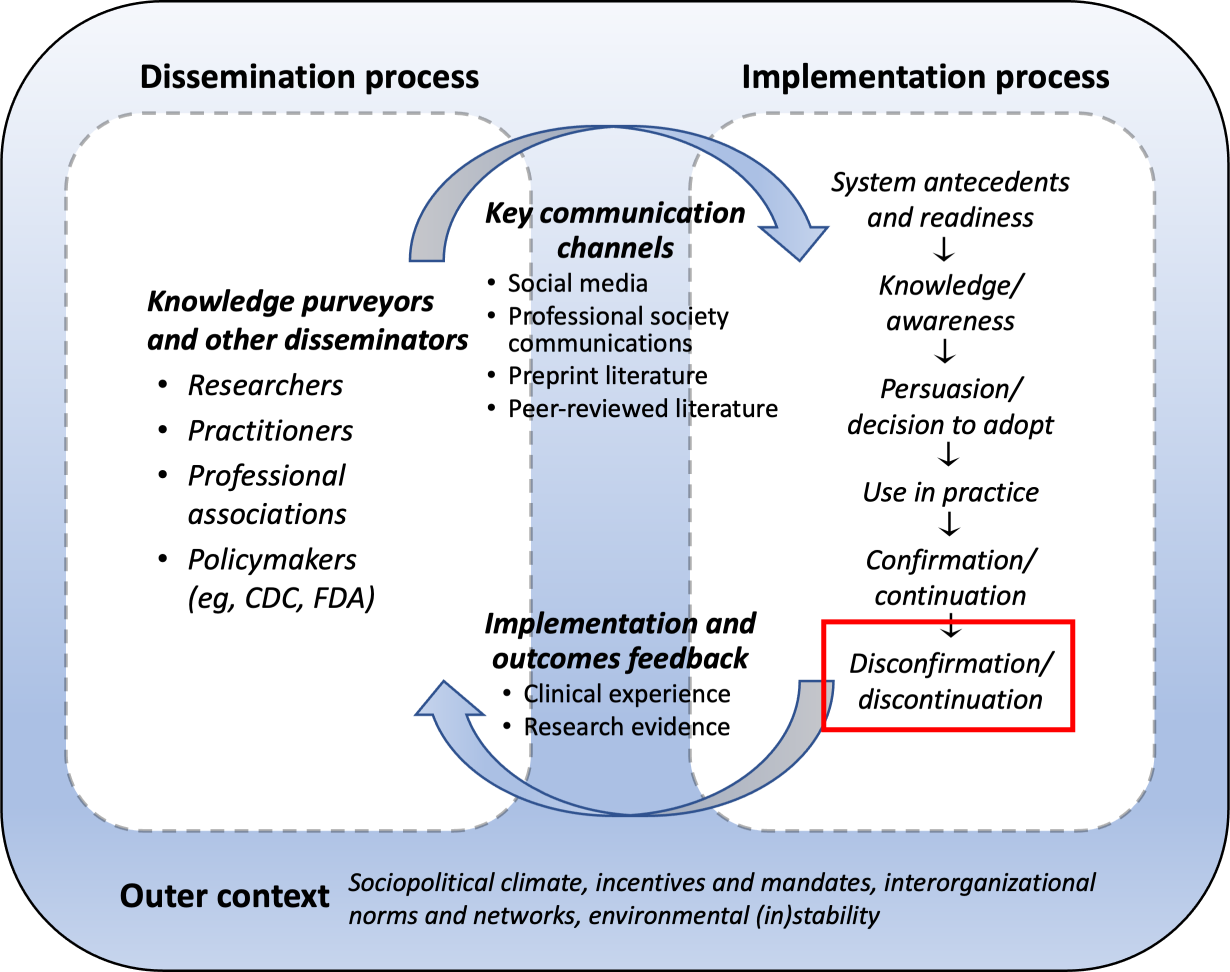
Diffusion framework for COVID-19-related emergency department innovations [11]. CDC: Centers for Disease Control and Prevention; FDA: US Food and Drug Administration.

Amid the public health emergency, ED clinicians learned about innovations (dissemination process) and decided whether and how to implement them (implementation process; see Figure 1). Many innovations were also adopted throughout the hospital in response to COVID-19, but a large number were first implemented in the ED. As new evidence emerged, there were changes in innovation adoption and use: some innovations were sustained, while others were discontinued. In response to emerging anecdotal and peer-reviewed evidence during the pandemic, clinicians discontinued practices that proved unnecessary, ineffective, or even dangerous. This process is called deimplementation. Deimplementation, or deadoption, is the process by which practices deemed harmful, ineffective, or no longer necessary are discontinued within an organization [8-10].

While the implementation of care innovations has been and continues to be well studied in the ED setting [12,13], less attention has been paid to deimplementation. Deimplementation differs from implementation in many important ways. A recent review noted that psychological factors and biases are more relevant to deimplementation than implementation, as many deimplementation activities involve stakeholders actively changing their behaviors [14,15]. Consequently, different behavioral change techniques are used in implementation and deimplementation studies, making the case that deimplementation should be studied as a separate process from implementation [16]. Compared to implementation, deimplementation seems to be hindered more greatly by local (ie, departmental) priorities and biases among leaders and practitioners within health systems, in addition to the traditional outside factors impacting implementation, such as social and economic barriers [14], likely due to the relative autonomy providers have in providing health services. Additionally, a narrative review of recent studies of embedded pragmatic clinical trials found that factors outside of effectiveness can determine deimplementation, such as perceived benefits, costs, and alignment with other policies or priorities [17]. However, information regarding factors that impact clinician decision-making around the deimplementation of innovations is limited [18], and even less is known about factors influencing deimplementation during a public health emergency, such as the COVID-19 pandemic. Further study of important processes and determinants for successful deimplementation is critical [19].

Attempts to formalize deimplementation in the context of implementation have linked both using 4 types of change for deimplementation: partial reduction, complete reversal, substitution with related replacement, and substitution with unrelated replacement of existing practices [20]. Unlike many previous deimplementation studies, these changes are usually related to deimplementing care processes rather than reducing the use of low-value treatments, such as improper medication delivery. In the context of the COVID-19 pandemic, this provides an opportunity to update and enhance preexisting deimplementation literature to focus on cases where the deimplementation involves the entire intervention, not just the degree or dose [21]. The synthesis model proposed by Niven et al [22] for the process of deadoption better aligns with deimplementation decision-making in the ED: identifying and prioritizing low-value practices is a key component of the model.

This study is part of a larger examination of the dissemination and implementation of care innovations during COVID-19. We utilized an adapted form of the Greenhalgh et al [11] framework for the diffusion of innovations in health services to systematically assess both implementation and deimplementation activities. In this paper, we examined factors relating to the deimplementation of innovations and the resulting deimplementation processes in the ED during the COVID-19 pandemic. The goal of this study was to understand how ED clinicians decided to discontinue using COVID-19 care innovations by identifying processes and factors that influenced decision-making.

## Methods

### Study Design and Setting

We conducted virtual pilot interviews and focus groups with ED clinicians to understand their experiences with practices related to the deimplementation of COVID-19 care innovations. The discussion guide was informed by a framework adapted from Greenhalgh et al [11] and modified after piloting with 1 physician, 1 nurse, and 1 nurse practitioner. After the initial pilot interviews, no changes were made to the content discussed or focus group guides. Thus, findings from the 3 pilot interviews were included in our overall analysis.

To meet our study objective, we conducted 13 subsequent focus group discussions with clinicians from 8 hospital-based EDs across the United States in 2022 and 2023. Because we believed that responses would differ by provider type and that professional hierarchy power dynamics could impact participant responses and comfort levels, we organized separate groups for ED prescribers (ie, physicians [medical doctors; MDs] and advanced practice providers [APPs]) and for other ED clinicians (ie, registered nurses [RNs] and respiratory therapists [RTs] at each hospital to facilitate deeper conversations and limit concerns about nonparticipation [23].

### Focus Group Recruitment

We used maximum diversity sampling to recruit personnel in hospital–based EDs to participate in focus groups [24]. Hospitals were diverse by US Census Bureau region (4 regions) [25], academic hospital status (binary), rurality (binary), and safety net status (binary), including one at the epicenter of the pandemic (New York City). A site-specific champion was recruited by email and assisted with recruitment and scheduling.

### Participant Recruitment and Data Collection

Focus group participants were recruited by email. Participants were eligible if they had experience providing care in a participating hospital’s ED during the COVID-19 pandemic. Forty-nine clinicians from 8 hospitals participated in the focus groups.

Focus groups lasted approximately 60 to 90 minutes and included at least 1 moderator and 1 notetaker. Three researchers (SF, CB, and CC) with both clinical and focus group experiences led the discussions, while other researchers (SH and NQ) took notes. Before each focus group, the moderator introduced themselves and provided a brief description of the research project. Focus group discussion topics included gaining information about COVID-19 treatment and sources of information, facilitators and barriers to adopting innovations, processes for confirming and sustaining promising innovations, processes for discontinuing innovations that were not promising, and how contextual factors impacted innovation implementation and deimplementation. Textbox 1 displays focus group questions specifically related to deimplementation, as discussed in this paper (for the full interview protocol, see Huilgol et al [26]). We continued conducting focus groups until thematic saturation was determined [27]. Study leaders assessed thematic saturation in team discussions after each focus group based on insights shared during the discussions.

Textbox 1. **Sample focus group questions.**

Deimplementing innovations

Sample questions:

- Tell me about a time you tried something and immediately said, I’m not doing that again? What was that like? What made you decide not to try it again?
- Here are some examples of things you might have stopped using: intubation boxes, reusing PPE, plasma, nasal steroids. Were any of these things you were doing and then stopped? Tell us about it. What made you make that decision?
- Can you give an example of one innovation you or your institution adopted and later rejected? Please describe how the decision was made to reject it. [Reject is defined as to stop using after having tried it.]
  - How did you personally decide whether to reject it?
  - How did your department or ED decide whether to reject it?
  - How did the institution as a whole decide to reject it?

### Ethical Considerations

This work was approved by the RAND Human Subjects Protection Committee (project 2021-N0714). Participants provided verbal consent at the start of the focus group and were compensated with a US $150 gift card for their time and participation. After receiving participant consent, focus groups were audio-recorded and transcribed for analysis. Participants’ privacy and confidentiality were protected by securely storing focus group transcripts and recordings, limiting access to authorized researchers, and de-identifying data prior to analysis to ensure no personal information was linked to participant responses.

### Data Analysis

We used both deductive and inductive analysis techniques to thematically code the focus group data [28]. We started by creating a preliminary codebook defined by topics included in the focus group discussion guide. We then used inductive processes to identify broader patterns and themes from the coded data. After the first focus group was completed, 2 researchers with expertise in health services and qualitative methodology (SH and NQ) independently reviewed a single transcript and applied codes to assess the quality of the codebook and the frequency of codes, representing approximately 10% of all coded data. The 2 coders performed an interrater reliability test and calculated a κ score of 0.72, indicating good agreement [29]. The codebook was updated and finalized based on this coding. As new themes and insights emerged during focus group discussions, the authors updated and added new codes to the codebook. Additions to the codebook were shared via regular weekly meetings among the project team [30].

The remaining transcripts were then divided in half and each coded by a single author. The same 2 authors met regularly to review coding and address any questions that emerged to ensure consistent code application. After coding was completed, the authors discussed coded excerpts holistically to identify general themes and subthemes that differed across groups. To ensure accuracy, one author reviewed a transcript coded by the other author at the beginning and end of the coding process.

The results are categorized by the first 2 phases of deadoption from the framework proposed by Niven: (1) identifying and prioritizing low-value clinical practices and (2) facilitating the deimplementation process. These 2 phases complemented the Greenhalgh framework guiding our analysis as they further explicate specific issues related to deimplementation [11]. All qualitative analyses were conducted in Dedoose [31]. We followed the COREQ (Consolidated Criteria for Reporting Qualitative Research) (Checklist 1) as a framework for data reporting [32]. Results about the implementation of innovations and information uptake in EDs have been published in detail elsewhere (see Huilgol et al [26] and Qureshi et al [33]).

## Results

### Hospital and Participant Characteristics

In addition to 3 pilot interviews, we conducted 13 semistructured focus group discussions. In total, 49 clinicians from 8 hospitals participated: 24 *prescribers* (17 ED physicians and 7 APPs) in a total of 7 focus groups, ranging from 3 to 6 participants per site focus group, and 25 *other clinicians* (18 RNs and 7 RTs) in a total of 6 focus groups, ranging from 3 to 6 participants per site focus group (Table 1). Fourteen additional clinicians were invited to participate in focus groups but declined, bringing our total response rate to 78%.

**Table 1.** Hospital and participant characteristics.

Hospital ID	Region	Focus group type				Total
		Physicians (MDs^a^)	APPs^b^	RNs^c^	RTs^d^	
Hospital 1	Midwest	6	0	5	0	11
Hospital 2	West	4	0	2	1	7
Hospital 3 (including 1 APP pilot)	West	3	1	5	2	11
Hospital 4	Northeast	2	1	1	4	8
Hospital 5	South	1	2	4	0	7
Hospital 6	South	0	3	0	0	3
Hospital 7 (pilot)	West	0	0	1	0	1
Hospital 8 (pilot)	West	1	0	0	0	1
All	National	17	7	18	7	49

a MD: medical doctor.

b APP: advanced practice provider.

c RN: registered nurse.

d RT: registered therapist.

Of the participating EDs, 4 EDs were in the West (3 Pacific and 1 Mountain), 2 were in the South (1 South Atlantic and 1 East South Central), 1 ED was in the Midwest (West North Central), and 1 ED was in the Northeast (Middle Atlantic). Some hospitals had 2 focus groups within their EDs, while others only had one. Overall, clinicians averaged 11.2 (SD 7.5) years of experience in their profession as ED clinicians (mean 9.4, SD 7.9 for physicians; mean 11, SD 4.3 for APPs; mean 12, SD 8.1 for RNs; and mean 14, SD 7.5 for RTs; Table 2). In addition, 67% (33/49) identified as female, 71% (35/49) identified as White, and 6% (3/49) identified as Hispanic.

During focus groups, we asked participants about examples of innovations they or their institutions adopted and later rejected. We asked them to describe how the decision was made to reject the use of the innovation at the *individual*, *departmental,* and *institutional* levels. Textbox 2 lists examples of discontinued COVID-19 care innovations mentioned by participants in focus groups, such as intubation boxes, plasma, PPE reuse protocols, and nasal steroids.

**Table 2.** Participant demographic characteristics.

Respondent type	Number of respondents	Average years of experience, mean (SD)
APP^a^	7	11 (4.3)
Female	5	11.8 (5.0)
Male	2	9 (1.4)
ED^b^ physician	17	9.4 (7.9)
Female	6	6.8 (5.7)
Male	11	10.8 (8.8)
Nurse	18	12 (8.1)
Female	16	11.4 (7.1)
Male	2	16.5 (17.7)
Respiratory therapist	7	14 (7.5)
Female	6	12.8 (7.5)
Male	1	21 (N/A)^c^
Grand total	49	11.2 (7.5)

a APP: advanced practice provider.

b ED: emergency department.

c N/A: not available.

Textbox 2. **Examples of discontinued COVID-19 care innovations.**

- Intubation boxes/shields
- Early intubation
- Plasma
- Deep cleaning COVID-19 patient rooms
- Varieties of COVID-19 tests
- V60 (ventilator type)
- PPE reuse protocols
- Nasal steroids and fluids

We present findings related to innovation deimplementation in Table 3, with subsections on factors and processes related to deimplementation. Tables3  4 highlight quotations that are representative of the major themes. We then identified the factors related to the deimplementation of COVID-19 care innovations that participants shared during focus groups.

**Table 3.** Identifying and prioritizing innovations for deimplementation: themes and illustrative quotations.

Themes	Illustrative quotation
Poor patient outcomes	*By the time we were regularly dealing with severely ill patients, it was evident from the experience—largely out of other [ED*^*a*^ *group] sites but also somewhat early in the publications—that early intubation actually turned out to be a really bad idea due to bad outcomes. So, we were actually I think early adopters of not [doing] early intubation in relation to our tertiary care receiving facilities.* -MD^b^, West
Little to no evidence of change	*I think it was a little bit of kind of seeing your patients with the plasma, it never really seemed to change anything. And there was also a lot of data coming out that was showing plasma, a) doesn’t really seem to work and even there’ve been a couple of studies that were showing harm with plasma…I think we kind of just were like, eh, it doesn’t really seem to be doing much and the patients it maybe gets some gains or it’s very minimal and so let’s just stop wasting our time with it*. -MD, Midwest
Burdensome	*I honestly don’t recall there being a discussion, like, oh, these [intubation boxes] are so cumbersome to use, we’re just not going to use them anymore. I don’t, I think we all probably just got comfortable with our PPE*^*c*^ *and I think we were pretty lucky. So maybe there are things that happened that I’m not aware of but it seems like we all did a really good job with PPE*. -MD, Midwest
Compliance	*I feel like we just realized nobody is using [telemedicine]. And that’s kind of how you know that you kind of stop that process. So I think that’s one, is just realizing that nobody is using that equipment or following those processes anymore. In the ED if it works people will usually stick with it or create a workaround as needed. But telemedicine is really, really hard for us to do*…. *-*RN^d^, West
Patient volume	*I think a lot of [discontinuing innovations] was if we were going to surge or not. So, they would have contingency plans, based on national predictions if things were going to surge or not. So they would be like, okay, we’re having lower volumes now, so we’re going to start discontinuing this. But in two weeks if it starts going back up, this is the order in which we’re going to start things back*. -MD, South
Availability	*Yeah, the only thing I stopped doing was double using the N95. Because now it’s available, so I don’t have to. And another thing we did was to...you know, they provided more supplies which was good, so you don’t have to reuse the N95. And you don’t have to reuse the surgical mask which is good. And it makes everyone feel more comfortable*. -APP^e^, South
Leadership	*I think one of our doctors has an ER throughput committee, and so that was brought up in that throughput committee. And he discussed it with infection prevention. And then was able through leadership to make that decision. Like, we [stopped deep cleaning rooms] before but it wasn’t official and then people were questioning it. So that’s when the committee got involved*. -RN, Midwest
New information	…*In the case of intubation boxes, it was a pain to use and then I think there was just like an abstract where they had done an airflow study and they showed how airflow happened and that it actually directed more viral particles back onto the [person performing the procedure] or something like that. And so then that was just the excuse that everyone needed to let us be like ‘Oh, thank God,’ and we got rid of that thing*. -MD, West

a ED: emergency department.

b MD: medical doctor.

c PPE: personal protective equipment.

d RN: registered nurse.

e APP: advanced practice provider.

**Table 4.** Processes related to the deimplementation of innovations: themes and illustrative quotations.

Themes	Illustrative quotation
Leadership guidance	*I remember that we were really counting on the guidance that we were getting from our leadership in terms of what the next, you know, what the next steps were, and then obviously they were using government resources and what not to pass that guidance on to us*. - APP^a^, Northeast
Collaboration among colleagues	*And so it all came down to…just [saying],* “*I think we should hold off” or* “*hey, I think we should talk to the ICU and see what they think about this because they’re the ones who are gonna be managing it later down the road.” And then ultimately that dialogue facilitated our own institutional changing in practice and how we approached intubation…it also evolved with the amount of information that we knew about COVID later on, saying that early intubation will not necessarily save someone’s life*. -MD^b^, West
No process/passive	*So I think that’s one, is just realizing that nobody is using that equipment or following those processes anymore. Some other things, like we were doing the antibodies for a while, those kind of treatment plans that you don’t really ever stop doing but you just realize that you don’t have a patient population that fits that requirement anymore. So I can’t think of a lot of things that we purposely stop. Things just kind of tend to run their course*. -RN^c^, West

a APP: advanced practice provider.

b MD: medical doctor.

c RN: registered nurse.

### Factors Contributing to Identifying and Prioritizing Innovations for Deimplementation

#### Clinician Experiences

During focus groups, multiple clinicians noted that the experience of observing poor patient outcomes was a common reason for discontinuing COVID-19 care innovations. For example, participants frequently mentioned early intubation as a deimplemented innovation: early in the pandemic, it was common practice to immediately intubate many patients affected by COVID-19 when they arrived at the ED as this practice was thought to be clinically beneficial. Several focus group participants shared that they stopped early intubation after their direct observation of poor outcomes. As one clinician elaborated:

*Yeah, so we did try early intubation; we were very aggressive about it. And then I think after we realized that early intubation wasn’t necessarily for temporary outcomes in patients who are on the ventilator for a long time and they get complications, learning from our ICU colleagues, and the challenges of the intubation itself are…in post-intubation. it was even harder to deal with, then we scaled it back….*

[MD, West]

Witnessing poor outcomes made clinicians reconsider whether it was useful to intervene early with intubation. One participant talked about how the “initial panic” of COVID-19 caused clinicians to initiate intubation early in care processes. The results of this intervention were not always beneficial for the patient. Another clinician highlighted how tracking outcomes and data were important in deciding whether to discontinue an innovation. Relatedly, if a care innovation showed little to no evidence of change in patient outcomes, it was stopped. For example, a participant mentioned that giving patients plasma—an innovation hoped to provide antibodies to sick patients from recovered patients—was an innovation that “never seemed to change anything,” with minimal gains. One ED clinician stated that giving plasma never seemed to make a difference for patients:

*…there was also a lot of data coming out that was showing plasma doesn’t really seem to work and even there’ve been a couple of studies that were showing harm with plasma…I think we kind of just were like, eh, it doesn’t really seem to be doing much and the patients it maybe gets some gains or it’s very minimal and so let’s just stop wasting our time with it.*

[MD, Midwest]

Additionally, intubation boxes were frequently brought up as a discontinued innovation because clinicians highlighted that they were cumbersome to use. As new evidence-based information became available, clinicians stopped using them.

#### Contextual Factors and Research Evidence

Participants reported that once they realized nobody was using the equipment or following procedures for certain innovations, those innovations were eventually discontinued. As one clinician stated, “the innovation[s] just ran [their] course.” For example, one clinician reported that telemedicine was an innovation that was never officially discontinued but was eventually stopped due to lack of use. One participant shared that decision-making around discontinuing innovations was based on whether there was a predicted surge or high patient volume. With lower patient volumes, clinicians would discontinue certain innovations, particularly those affecting triage or physical management. Conversely, if they were predicting a high patient volume or a surge, they would restart certain innovations (eg, implementing a surge schedule with overnight coverage).

Relatedly, the availability of the innovation mattered as well. One prominent example was related to reusing PPE. Once there were adequate supplies of N95 masks available, participants shared that they stopped reusing the PPE:

*Yeah, the only thing I stopped doing was double using the N95. Because now it’s available, so I don’t have to. And another thing we did was to...you know, they provided more supplies which was good, so you don’t have to reuse the N95. And you don’t have to reuse the surgical mask which is good. And it makes everyone feel more comfortable*.

[APP, South]

Leadership also played a significant role in identifying and prioritizing discontinued innovations. Across focus groups, participants mentioned that directions regarding innovation deimplementation came from leadership guidance or hospital-wide committees. Oftentimes, official guidance from leadership teams or committees legitimized deimplementation decisions:

*I think one of our doctors has an ER throughput committee, and so that was brought up in that throughput committee. And he discussed it with infection prevention. And then was able through leadership to make that decision. Like, we [stopped deep cleaning rooms] before but it wasn’t official and then people were questioning it. So that’s when the committee got involved*.

[RN, Midwest]

Finally, as research evidence on different care processes and new innovations emerged, such as high-flow oxygen, clinicians realized that they were able to disregard early intubation and its associated risks and replace it with other modalities. A clinician noted that utilizing other innovations led to the deimplementation of early intubation:

…*as time went by, we started to use high flow [oxygen], we started other modalities and were able to avoid [early] intubation*.

[RT, Northeast]

Early intubation quickly ceased to be viewed as a standard of care and was described as a discontinued innovation by multiple focus groups.

### Processes Related to the Deimplementation of Innovations

#### Leadership Guidance

The process of deimplementing innovations occasionally included some sort of leadership or committee. The decisions were made and applied hospital-wide, usually in tandem with Centers for Disease Control and Prevention guidance or other government resources. As one participant shared, “We had a couple APPs who started using [ivermectin] in the beginning. And then once we got some leadership direction, it stopped. But we had a couple that did.” Another clinician underscored the importance of leadership for guidance during the process of discontinuing innovations:

*I remember that we were really counting on the guidance that we were getting from our leadership in terms of what the next steps were…*

[APP, Northeast]

#### Collaboration Among Colleagues

Collaboration between hospitalists and colleagues was another commonly discussed topic during focus groups when asked about deimplementation. For example, one clinician stated:

*[with] every COVID patient, we were really thinking out loud. We were just kind of having open conversations about disposition for each of them, just because it wasn’t standardized. It wasn’t like a kidney stone that we kind of knew the process. I think every patient was truly thought out loud and it was really kind of a team-based decision*.

[APP, South]

Additionally, during early intubation, clinicians shared how they would discuss the process with other clinicians in the intensive care unit and determined what worked best for the patient. A clinician described how the approach of early intubation evolved and was eventually discontinued—the process involved multiple factors, such as discussing with colleagues in the intensive care unit, sharing information, and examining outcomes:

*And so it all came down to…just [saying]…* “*Hey, I think we should talk to the ICU and see what they think about this because they’re the ones who are going to be managing it later down the road.” And then ultimately that dialogue facilitated our own institutional changing in practice…it also evolved with the amount of information that we knew about COVID later on, saying that early intubation will not necessarily save someone’s life*.

[MD, West]

#### No Process/Passive Deimplementation

Sometimes, however, there was no process to officially deimplement innovations; it was passive. One participant shared that they stopped using monoclonal antibodies because the patient population for it had declined. The clinician stated,

*So I think that’s one, is just realizing that nobody is using that equipment or following those processes anymore…Some other things, like we were doing the antibodies for a while, those kind of treatment plans that you don’t really ever stop doing but you just realize that you don’t have a patient population that fits that requirement anymore. So I can’t think of a lot of things that we purposely stop.*

[RN, West]

In these cases, it seems that enough people simply stop doing something, without any central decision or authority, to the point that it is no longer used.

## Discussion

### Principal Findings

While there have been calls to identify and reduce low-value care before [18,33,34] and during [35] the COVID-19 pandemic, this study adds to what is known about innovation deimplementation during the pandemic by characterizing the factors and processes that led ED clinicians to decide to discontinue using COVID-19 care innovations. Overall, clinicians highlighted that patient outcomes, collaboration and discussion with ED colleagues, supply availability, and protocols from leadership as factors that contributed heavily to decisions about sustaining or deimplementing innovations in EDs during the COVID-19 pandemic.

Prior studies show that, in routine conditions, enablers of effective low-value care deimplementation include leadership commitment, provider engagement, provider training, performance feedback to providers, and shared decision-making with patients [18,35]. Under usual conditions, deimplementation involves strong external support, provider engagement, and training and education to impact knowledge and behavior; therefore, it is typically a slow process [36]. Fundamental to its goals, the deimplementation of health services involves engaging with and changing the behavior of health care providers. A recent systematic review of determinants of deimplementation found that health care provider knowledge, expectations, attitudes, and behaviors are key determinants of deimplementation [37]. In addition, a central concern of health care providers is the trust that the deimplementation effort is necessary and will not harm patients currently receiving a low-value treatment, while also improving patient health in the long term. This requires building support and buy-in for deimplementation from front-line staff [38]. Clinicians need both motivation and support to properly facilitate deimplementation. Our findings confirm the importance of leadership, collaboration, and discussion with ED colleagues for the deimplementation of newly adopted innovations.

However, the speeds and processes for innovation deimplementation differ significantly during a public health emergency compared to more stable time periods in terms of scientific discoveries related to health. For example, hydroxychloroquine was once thought to be potentially helpful before evidence ultimately discouraged its use. Bradley et al [39] examined hydroxychloroquine deimplementation and found that the proportion of hospitalized patients receiving hydroxychloroquine in 2020 decreased drastically over the span of a few months (55.2% in March and April, 4.8% in May and June, and 0.8% in July and August). The authors hypothesize that this was because hydroxychloroquine was not deeply entrenched in hospital practice; the deimplementation of long-standing innovations may be more challenging due to the sunk cost fallacy and loss aversion [40]. Clinicians may have been more amenable to rapid deimplementation due to awareness of their own limited knowledge, along with the limited evidence-based information and the rapidly changing information during the pandemic, the latter of which was a factor related to innovation deimplementation that we identified during focus groups.

Although there are some conceptual frameworks and criteria for innovation and intervention deimplementation, they may not adequately describe the factors affecting deimplementation during rapidly evolving situations, such as the COVID-19 pandemic [22]. Much of the deimplementation literature focuses on stopping long-standing low-value care, whereas during the pandemic, many discontinued practices were short-term and context-dependent as new evidence emerged [9,10,18,33]. The pace and timing of discontinuing innovations in ED settings during COVID-19 was a major distinguishing factor. The 3 criteria posed by McKay et al [9] to identify if interventions are appropriate for deimplementation (ie, interventions that are “not effective or harmful,” “not the most effective or efficient to provide,” and “that are no longer necessary”) also may fall short in describing conditions during the COVID-19 pandemic because decisions about innovations had to be made in the absence of evidence-based information about innovation efficacy, harm, and efficiency.

Overall, innovation deimplementation in ED settings during the COVID-19 pandemic was different from typical deimplementations due to a lack of information as well as the rapid evolution of information in response to emerging and dynamic environments. Clinicians tended to discontinue the innovation if leadership support, evidence-based information, funding, or resource availability were limited. We also found differences between innovations that were actively stopped due to poor outcomes and new evidence emerging, such as early intubation and intubation boxes, and those that were stopped due to passive processes or a lack of necessity. Prior deimplementation models and frameworks do not account for circumstances where deimplementation is unintentional. Passive deimplementation of innovations may require additional clinician autonomy and greater flexibility, whereas active deimplementation involves highly formalized and structured pathways. Active deimplementation is more difficult compared to passive deimplementation because passive deimplementation occurs without follow-up and drastic changes to practice.

These findings have implications for public health emergency situations. Our focus groups suggest that institutions will be most able to quickly adopt and deimplement innovations informed by local conditions, such as leadership, patient volume, replacement, new information, compliance, and availability, as described above. While prior research identified several other factors that are linked to deimplementation, such as fear of medical malpractice, revenue, competitive advantage, and liability [18], those factors were less relevant in the high-pressure fast-paced environment at the beginning of the pandemic. Our findings also show that factors around deimplementation during a public health emergency were highly localized and akin to a pilot-testing approach—innovations were often hospital-specific and performed at a local scale. Using limited knowledge, clinicians were willing to try and adopt innovations rapidly within their organizations, and the level of evidence needed to implement the innovations was much lower. However, when new evidence-based information became available, clinicians also had to be willing to deimplement those tested innovations quickly. Future public health emergencies will require rapid evaluation and will likely be driven by a narrower group of factors than during normal times.

At the same time, as seen through the Greenhalgh framework, overarching factors that are important for implementation and deimplementation in normal times are still needed during public health emergencies, such as stakeholder engagement, leadership buy-in, and organizational readiness [11,36]. In the future, we should prepare for and embrace the adoption of innovations with limited data, with the willingness to easily deimplement as those data appear and with the knowledge that the deimplementation of innovations used for shorter periods of time may be easier [38,40,41].

### Limitations

Because our focus groups were conducted 2 to 3 years after March 2020, recall bias may have influenced ED participants’ self-reported experiences. Additionally, despite recruiting from a diverse set of EDs, our sample is not nationally representative, and we do not have information on those who declined to participate in the focus groups. We also do not believe our results are generalizable, but they have been influential in informing the development of a nationwide survey that will be administered to ED physicians and nurses about implementation and deimplementation during the COVID-19 pandemic. Some of the focus groups were coded by only 1 coder, but the interrater reliability of dually coded transcripts was high [42]. Finally, at the time of writing this article, many COVID-19 care innovations implemented and deimplemented in the early stages of the pandemic now have more evidence to support their benefit or lack thereof.

### Conclusion

ED clinicians are critical to the response to disease outbreaks, such as the COVID-19 pandemic. During uncertain times, clinicians must rapidly implement new protocols and practices and assess these changes in real time under uncertain and potentially dangerous conditions. Stopping unhelpful or harmful practices appropriately is equally important. This study is unique because it examined sustainability and deimplementation during a time of uncertainty and identified unique factors and processes that affect the decision to deimplement innovations, such as poor outcomes, patient volume, availability, and leadership support, when trying to choose the best approach to treating an acute and serious disease.

## Supplementary material

10.2196/82088 Checklist 1 COREQ checklist.

## Abbreviations

APP: advanced practice provider
COREQ: Consolidated Criteria for Reporting Qualitative Research
ED: emergency department
MD: medical doctor
PPE: personal protective equipment
RN: registered nurse
RT: respiratory therapist
