# Supplementary material for: Innovation Deimplementation in Emergency Departments During the COVID-19 Pandemic: Qualitative Study of Clinicians’ Decision-Making
Source: Interact J Med Res. 2026 May 22;15:e82088. doi: 10.2196/82088 (PMC13197154; doi:10.2196/82088)
Supplement: Checklist 1 [file ijmr-v15-e82088-s001.pdf]

Innovation de-implementation in emergency departments during the COVID-19 pandemic: A qualitative study of clinician decision-making

Supplement 1. COREQ Checklist

In the table below, we describe how our study addresses the domains and subdomains on the COREQ checklist.

	**Guide Questions/Description**	**Page Number (if applicable)**	**Relevant Text (or other details)**
**Domain 1: Research team and reflexivity**			
Personal Characteristics			
1. Interviewer/ facilitator	Which author/s conducted the interview or focus group?	7	"Three researchers (SF, CB, CC) with both clinical and focus group experience led the discussions with another researcher (SH, NQ) taking notes."
2. Credentials	What were the researcher's credentials? e.g., PhD, MD	1	
3. Occupation	What was their occupation at the time of the study?	7,8	"Three researchers (SF, CB, CC) with both clinical and focus group experience led the discussions with another researcher (SH, NQ) taking notes."<br><br>"After the first focus group was completed, two researchers with expertise in health services and qualitative methodology (SH, NQ)…"
4. Gender	Was the researcher male or female?	N/A	Our research team included both males and females.

5. Experience and training	What experience or training did the researcher have?	7, 8	“Three researchers (SF, CB, CC) with both clinical and focus group experience led the discussions with another researcher (SH, NQ) taking notes.”<br><br>“After the first focus group was completed, two researchers with expertise in health services and qualitative methodology (SH, NQ)…”
Relationship with participants			
6. Relationship established	Was a relationship established prior to study commencement?	6	Participating ED sites and participants had no prior relationship with the study team.<br><br>A site-specific champion was recruited by email, and this individual assisted in identifying dates and times likely to be well-attended by participants at their site.<br><br>“A site-specific champion was recruited by email and assisted in recruitment and scheduling.”
7. Participant knowledge of the interviewer	What did the participants know about the researcher? e.g., personal goals, reasons for doing the research	7	“Before each focus group, the moderator introduced themselves and provided a brief description of the research project.”
8. Interviewer characteristics	What characteristics were reported about the interviewer/facilitator? e.g. ,bias, assumptions, reasons and interests in the research topic	7	“Before each focus group, the moderator introduced themselves and provided a brief description of the research project.”
**Domain 2: Study design**			

Theoretical framework			
9. Methodological orientation and Theory	What methodological orientation was stated to underpin the study? E.g., grounded theory, discourse analysis, ethnography, phenomenology, content analysis	8-9	We used both deductive and inductive analysis techniques to thematically code of focus group data.27 We started by creating a preliminary codebook defined by topics included in the focus group discussion guide, based on the Greenhalgh framework.22 We then used inductive processes to identify broader patterns and themes from the coded data. After the first focus group was completed, two researchers with expertise in health services and qualitative methodology (SH, NQ) independently reviewed a single transcript and applied codes to assess the quality of the codebook and the frequency of codes. The two coders performed an inter-rater reliability test and calculated a kappa score of 0.72, indicating good agreement.28 The codebook was updated and finalized based on this coding. As new themes and insights emerged during focus group discussions, coders updated and added new codes to the codebook. Additions to the codebook were shared via regular weekly meetings among the project team.29<br><br>The remaining transcripts were then divided in half and each coded by a single coder. The two coders met regularly to review coding and address any questions that emerged to ensure consistent coding practice. After coding was completed, the coders discussed coded excerpts holistically to identify general themes, and subthemes that differed across groups. To ensure accuracy, one coder reviewed a transcript coded by other coder at the beginning and end of the coding process. All qualitative analysis was conducted in Dedoose.30 We followed the Consolidated

			Criteria for Reporting Qualitative Research (COREQ) as a framework for data reporting.31 Results about implementation of innovations have been published in detail elsewhere (see Huilgol et al. 202426 and Qureshi et al. 202432); this manuscript focuses on de-implementation of recently adopted innovations.
Participant selection			
Sampling	How were participants selected? e.g., purposive, convenience, consecutive, snowball?	6, 7	"We used maximum diversity sampling to recruit personnel in hospital-based EDs to participate in focus groups [21]. Hospitals were diverse by US Census Bureau region (four regions) [22], academic hospital (binary), rurality (binary), and safety net status (binary). A site-specific champion was recruited by email and assisted in recruitment and scheduling."<br><br>"Participants were eligible if they had experience providing care in a participating hospital's ED during the COVID-19 pandemic."
Method of Approach	How were participants approached? e.g. face-to-face, telephone, mail, email?	6-7	"A site-specific champion was recruited by email and assisted in recruitment and scheduling. Focus group participants were recruited by email for either a physician/advanced practice providers (APPs) focus group or a registered nurses (RNs)/respiratory therapist (RTs) focus group."
Sample Size	How many participants were in the study?	9	"In addition to three pilot interviews, we conducted 13 semi-structured focus group discussions. In total, 49 clinicians from eight hospitals participated: 24 prescribers (17 ED physicians and seven APPs) in a total of seven

			focus groups, ranging from 3–6 participants per site focus group; and 25 other clinicians (18 RNs and seven RTs) in a total of six focus groups, ranging from 3–6 participants per site focus group (see Table 2).”<br><br>Of the participating EDs, four EDs were in the West (three Pacific and one Mountain), two were in the South (one South Atlantic and one East South Central), one was in the Midwest (West North Central) and one was in the Northeast (Middle Atlantic). Some hospitals had two focus groups within their EDs, while others only had one.”
Non-participation	How many people refused to participate or dropped out? Reasons?	N/A	Although there were scheduling issues for some focus groups, once participants logged on the Zoom meeting, they did not leave the meeting until the discussion concluded.
Setting			
Setting of data collection	Where was the data collected? e.g., home, clinic, workplace	7	“After receiving participant consent, we audio-recorded discussions on Zoom.gov for transcription.”<br><br>Participants in one focus group gathered at work for the virtual call. In all other focus groups, participants joined separately on their own.
Presence of non-participants	Was anyone else present besides the participants and researchers?	N/A	Nobody was present in the focus group discussion aside from participants and the research team.

Description of sample	What are the important characteristics of the sample? e.g., demographic data, date	9-10	Tables 2 and 3
Data Collection			
Interview Guide	Were questions, prompts, guides provided by the authors? Was it pilot tested?	6	We conducted pilot interviews and focus groups with ED clinicians to understand their experience with practices related to de-implementation of COVID-19 care innovations. The discussion guide was informed by a framework adapted from Greenhalgh et al., 200422 and modified after piloting with one physician, one nurse, and one nurse practitioner. After the initial pilot interviews, no changes were made to the content discussed or focus group guides. Thus, findings from the three pilot interviews were included in our overall analysis.
Repeat Interviews	Were repeat interviews carried out? If yes, how many?	N/A	There were no repeat focus groups carried out.
Audio/Visual Recording	Did the research use audio or visual recording to collect the data?	7	"After receiving participant consent, we audio-recorded discussions on Zoom.gov for transcription."
Field Notes	Were field notes made during and/or after the interview or focus group?	7	"Focus groups lasted approximately 60–90 minutes and included at least one moderator and one notetaker. Three researchers (SF, CB, CC) with both clinical and focus group experience led the discussions with another researcher (SH, NQ) taking notes."

Duration	What was the duration of the inter views or focus group?	7	"Focus groups lasted approximately 60–90 minutes and included at least one moderator and one notetaker. Three researchers (SF, CB, CC) with both clinical and focus group experience led the discussions with another researcher (SH, NQ) taking notes."
Data Saturation	Was data saturation discussed?	8-9	We used both deductive and inductive analysis techniques to thematically code of focus group data.27 We started by creating a preliminary codebook defined by topics included in the focus group discussion guide, based on the Greenhalgh framework.22 We then used inductive processes to identify broader patterns and themes from the coded data. After the first focus group was completed, two researchers with expertise in health services and qualitative methodology (SH, NQ) independently reviewed a single transcript and applied codes to assess the quality of the codebook and the frequency of codes. The two coders performed an inter-rater reliability test and calculated a kappa score of 0.72, indicating good agreement.28 The codebook was updated and finalized based on this coding. As new themes and insights emerged during focus group discussions, coders updated and added new codes to the codebook. Additions to the codebook were shared via regular weekly meetings among the project team.29<br><br>The remaining transcripts were then divided in half and each coded by a single coder. The two coders met regularly to review coding and address any questions that emerged to ensure consistent coding practice. After coding was completed, the coders discussed coded excerpts holistically to identify general themes, and subthemes that differed across groups. To ensure accuracy, one coder reviewed a transcript coded by other coder at the beginning and end of the coding process. All qualitative analysis was

			conducted in Dedoose.30 We followed the Consolidated Criteria for Reporting Qualitative Research (COREQ) as a framework for data reporting.31 Results about implementation of innovations have been published in detail elsewhere (see Huilgol et al. 202426 and Qureshi et al. 202432); this manuscript focuses on de-implementation of recently adopted innovations.
Transcripts Returned	Were transcripts returned to participants for comment and/or correction?	N/A	Transcripts were not released outside the research team to protect participant confidentiality and privacy.
**Domain 3: analysis and findings**			
Data analysis			
Number of data coders	How many data coders coded the data?	8	"…two researchers with expertise in health services and qualitative methodology (SH, NQ) independently reviewed a single transcript and applied codes to assess the quality of the codebook and the frequency of codes."
Description of the coding tree	Did authors provide a description of the coding tree?	7-9	See Table 1.<br><br>The remaining transcripts were then divided in half and each coded by a single coder. The two coders met regularly to review coding and address any questions that emerged to ensure consistent coding practice. After coding was completed, the coders discussed coded excerpts holistically to identify general themes, and subthemes that differed across groups. To ensure accuracy, one coder

			reviewed a transcript coded by other coder at the beginning and end of the coding process. All qualitative analysis was conducted in Dedoose.30 We followed the Consolidated Criteria for Reporting Qualitative Research (COREQ) as a framework for data reporting.31 Results about implementation of innovations have been published in detail elsewhere (see Huilgol et al. 202426 and Qureshi et al. 202432); this manuscript focuses on de-implementation of recently adopted innovations.
Derivation of themes	Were themes identified in advance or derived from the data?	8-9	We used both deductive and inductive analysis techniques to thematically code of focus group data.27 We started by creating a preliminary codebook defined by topics included in the focus group discussion guide, based on the Greenhalgh framework.22 We then used inductive processes to identify broader patterns and themes from the coded data. After the first focus group was completed, two researchers with expertise in health services and qualitative methodology (SH, NQ) independently reviewed a single transcript and applied codes to assess the quality of the codebook and the frequency of codes. The two coders performed an inter-rater reliability test and calculated a kappa score of 0.72, indicating good agreement.28 The codebook was updated and finalized based on this coding. As new themes and insights emerged during focus group discussions, coders updated and added new codes to the codebook. Additions to the codebook were shared via regular weekly meetings among the project team.29
Software	What software, if applicable, was used to manage the data?	9	“All qualitative analysis was conducted in Dedoose.”

Participant checking	Did participants provide feedback on the findings?	N/A	Participants did not provide feedback on the findings. During focus group discussions, the moderator clarified important points and restated them back to participants, to ensure data collection and interpretation was accurate.
Reporting			
Quotations presented	Were participant quotations presented to illustrate the themes/findings? Was each quotation identified? e.g., participant number	10-15, 20-21 (table 4, table 5)	Participant quotations were presented in text and in Table 4 and Table 5
Data and findings consistent	Was there consistency between the data presented and the findings?	10-15, 20-21 (table 4, table 5)	[see results section].
Clarity of major themes	Were major themes clearly presented in the findings?	10, 20-21 (table 4, table 5)	"We present findings related to innovation de-implementation in Table 5, with subsections on factors and processes related to de-implementation. Table 5 highlights quotations that are representative of the major themes. [Insert Table 5]"
Clarity of minor themes	Is there a description of diverse cases or discussion of minor themes?	10-15, 20-21 (table 4, table 5)	We have included subthemes under each major theme in the findings with quotations when particularly salient.
